# Post-Ebola Awakening: Urgent Call for Investing in Maintaining Effective Preparedness Capacities at the National and Regional Levels in Sub-Saharan Africa

**DOI:** 10.24248/EAHRJ-D-19-00019

**Published:** 2019-07-30

**Authors:** Serge Nzietchueng, Dominic Kambarage, Innocent B Rwego, Sayoki G Mfinanga, Anthony Mbonye, David Mutonga, Winyi Kaboyo, Issa Makumbi, Samuel Muriuki, Ndongo Casimir, Stephen Mduma, Charles Makasi, Andrew Y Kitua

**Affiliations:** a One Health Division, Department of Veterinary Population Medicine, College of Veterinary Medicine, University of Minnesota, St. Paul, MN, USA; b USAID/EPT-2 Preparedness and Response Project; c Mwalimu Julius Kambarage Nyerere University, Musoma, Tanzania; d Department of Biosecurity, Ecosystems and Veterinary Public Health, Makerere University, Kampala, Uganda; e National Institute for Medical Research, Dar es Salaam, Tanzania; f Department of Public Health, Muhimbili University of Health and Allied Sciences, Dar es Salaam, Tanzania; g Afrique One-ASPIRE; h College of Health Sciences, Makerere University, Kampala, Uganda; i Veterinary Service, Ministry of Livestock, Fisheries and Animal Industries; j National Public Health Institute, Abidjan, Côte d'Ivoire; k Public Health and Environmental Advancement Interventions “NGALAKERI” NGO, Morogoro, Tanzania

## Abstract

**Background:**

The 2014 Ebola outbreak reminded us of the importance of preparedness for addressing health security threats. Learning from this experience, we aim to (1) enhance the understanding of preparedness by policy and decision makers, (2) discuss opportunities for Africa to invest in the prevention of health security threats, (3) highlight the value of investing in preventing health security threats, and (4) propose innovations to enhance investments for the prevention or containment of health security threats at the source.

**Methods:**

We used observations of governments' attitudes towards investing in preparedness for health security prevention or containment at the source. We conducted a literature review through PubMed, the World Wide Web, and Mendeley using the keywords: “health emergency financing”, “investing in health threats prevention”, and “stopping outbreaks at the source”.

**Results:**

Countries in sub-Saharan Africa invest inadequately towards building and maintaining critical capacities for preventing, detecting, and containing outbreaks at the source. Global health security emergency funding schemes target responses to outbreaks but neglect their prevention. Governments are not absorbing and maintaining adequately capacity built through GHS, World Bank, and development aid projects – a lost opportunity for building and retaining outbreak prevention capacity.

**Recommendations:**

Governments should (1) allocate adequate national budgets for health honouring the Abuja and related commitments; (2) own and maintain capacities developed through International Development Aids, OH networks, research consortia and projects; (3) establish a regional health security threats prevention fund. The global community and scientists should (1) consider broadening existing health emergency funds to finance the prevention and containment outbreaks at the source and (2) Strengthen economic analyses and case studies as incentives for governments' budget allocations to prevent health security threats.

## INTRODUCTION

The 2014 Ebola outbreak shock prompted the establishment of the Global Health Security Agenda (GHSA). It exposed major health systems weaknesses, common to sub-Saharan African countries, including severe shortages of skilled health workers and gross systems unpreparedness to prevent, detect, and contain outbreaks at the source. Fear and panic due to unpreparedness were compounded by superstition, perceptions and practices among affected populations impeded effective disease containment policies including safe burial.*^1-5^*

We have observed that major huddles to systems strengthening arise from misconceptions and insufficient understanding of the full spectrum of activities necessary for effective preparedness against health threats among policy and decision makers, and unappreciation of the value of investing in long-term systems strengthening rather than short term interventions with immediate results.

We provide this opinion piece to provoke rethinking and encourage Policy and decision makers to value and invest in preparedness for health threats.

## UNDERSTANDING PREPAREDNESS FOR OUTBREAKS

**FIGURE 1.**
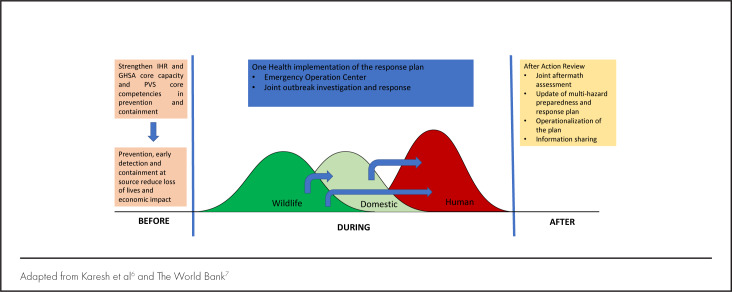
The Before-, During-, and After-Event Periods and Related Activities

Natural or human environmental disturbances may trigger spillover of pathogens from the wild to domestic life and eventually to human beings.^6,7^ Failure to rapidly and adequately detect and respond to such spilllover can lead to pathogen amplification in animal and human populations, resulting in uncontainable epidemics or pandemics. This spectrum can be categorised as “before”, “during”, and “after” outbreak periods. Figure 1 summarises the capacities requirements for each period.

## THE “BEFORE-OUTBREAK” PERIOD

The “before-outbreak” period is a peaceful time, which makes it optimal for strengthening capacities and collaborations among the human, animal, and environmental health systems to prepare to prevent or contain any outbreak at the source as provided by International Health Regulations (IHR) and World Organization for Animal Health (OIE) Standards on the performance of veterinary services (PVS).^8,9^

Essential capacities, as summarised in figure 1 (arrows showing pathogen spillover) include disease surveillance and detection, risk assessment and prediction, developing and implementing risk reduction strategies and interventions, mapping and monitoring risk behaviour in response to preventive interventions over time.^1,2^ This is the best period for multisectoral coordination and collaboration to assess, build, and maintain a vibrant mixed disciplines and skills workforce, enhance syndromic surveillance capacity for real-time reporting, and establish joint activities among animal and human health workers teams, hence strengthening multisectoral coordination and collaboration.^7,10,12^ It offers the opportunity to engage communities (traditional and spiritual leaders) and anthropologists to build community trust and strengthen community participation to avoid the kind of mistrust, beliefs and practices fueling outbreaks as evidenced during the 2014 Ebola outbreak.^11^

It is optimal for engaging the private sector to learn from their best practices, including regular simulation exercises in order to prevent or minimise the negative economic consequences of outbreaks on business, like what happened with Ebola, SARS and previous pandemics.^13-15^

Engaging national finance ministries and departments responsible for national budget allocations, to identify needs, and generate or revise annual national preparedness plans and strategies during this period will enhance their appreciation of the value of prevention before an outbreak especially if presented with economic analyses that highlight the size of potential savings is essential.^16-19^

## “DURING OUTBREAK” PERIOD

Usually, human case reporting to national health authorities and the World Health Organization (WHO), triggers national and global response and allows access to global emergency funds, while wildlife events are often neglected.

A well-prepared workforce (sufficient in skills and numbers), updated response plan, standard operating procedures (SOPs) and protocols for field epidemiology and laboratory investigations, case identification, management, and contact tracing are necessary during this period. A functional Emergency Operations Centers (EOC) is required to provide responsible leadership with clear lines of command and daily reporting of actions and behaviour of the outbreak. There is growing evidence that good multisectoral coordination optimises mobilisation and use of resources; enhance efficiency, transparency, accountability and the application of effective national and regional policies that minimise negative economic impact.^20-23^

## THE “AFTER-OUTBREAK” PERIOD

The “after-outbreak” period is for conducting thorough multisectoral After-Action Reviews (AAR) to determine the strengths of the response and identify related capacity gaps. Findings and lessons learnt are useful for reviewing the preparedness and response plans, response protocols, SOPs and strengthening the operational systems, for future responses. AAR further identifies important research gaps and informs on the effects of existing national and regional policies, guiding their revision.

## VALUE OF INVESTING IN PREVENTION AND EARLY DETECTION

There is unequivocal evidence in human and veterinary medicine that prevention is better than cure/response and strategies for disease prevention have been defined.^24^ Vacci-nation programmes have provided cost-effective disease prevention, eradication and significantly reduced related death and disabilities.^25-28^ Unfortunately, African governments invest inadequately towards building preventative capacities and implementing preventive interventions during the “before-outbreak” period since the results of this investment are not immediate and visible. Achieving the Abuja declaration goal of 15% allocation for health has been slow.^29-31^ Consequently, infrastructure for disease prevention, including laboratories, epidemiological surveillance and research receive inadequate African financial support and heavily rely on external financing. Routine disease prevention receives negligible budgets, and there are far too few health workers in Africa to efficiently and adequately manage the collective burden of illness and injury.^1,4,29-31^

**FIGURE 2.**
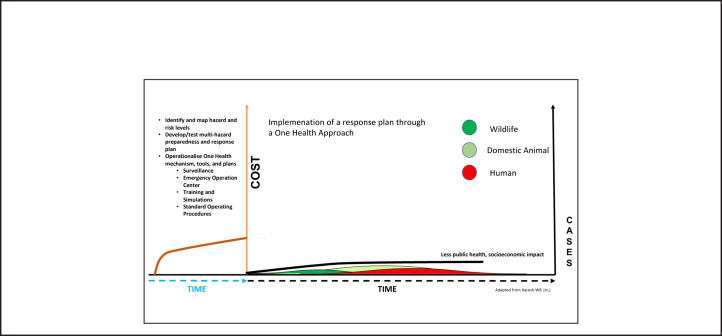
Active Routine Prevention, Detection, and Response Reduce Outbreak Size and Associated Social and Economic Costs

Effective surveillance and early warning systems operating routinely during the “before event” period (Figure 2 brown line) will trigger early actions preventing or containing outbreaks at the source (Figure 2 black line), reducing the sizes and impact of the outbreak considerably in wildlife (green), domestic animals (yellow) and humans (red). Related social and economic costs become minimal and manageable, as indicated by the black line in Figure 2.

**FIGURE 3.**
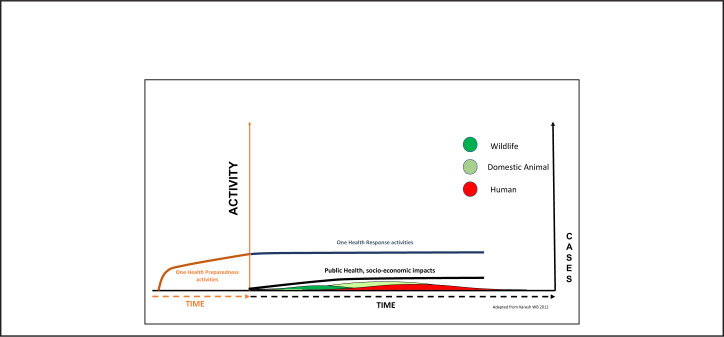
Sustained Routine Prevention and Detection Results in Minute Outbreaks and Governments' Savings

**FIGURE 4.**
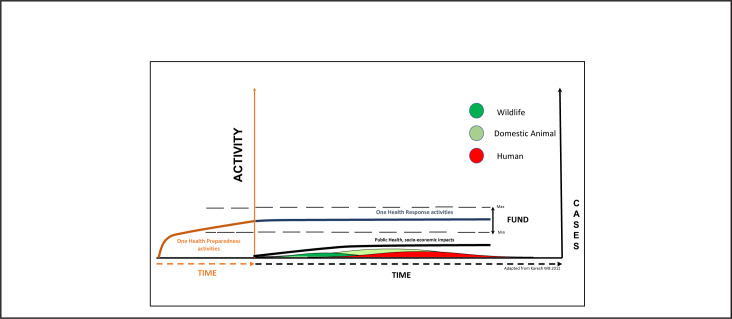
Funding to Maintain Effective Preparedness, Prevention, Detection, and Response Activities

Figure 3 shows a hypothetical situation where the routine cost of continuous One Health preparedness actions (brown and blue continuation) allows prompt detection and containment at the source resulting in less public health and socioeconomic impact (black line). In Figure 4, we hypothesise that the amount of money required to invest and maintain a vibrant prepared workforce and related infrastructure will fluctuate between a minimum during peacetime (dotted lower line) and a maximum during outbreaks (upper dotted line) but remain within limits affordable to national budgets. Investing in routine preparedness will enable countries to reduce epidemics to miniature events (Figure 3 and 4 green, yellow and red). Maintenance of the vibrant prepared work-force and systems' infrastructure will, therefore, strengthen the resilience of health systems against health threats.

Uganda is exemplary in that it has suffered 4 outbreaks between January and December 2017, which could have amplified into epidemic levels threatening the East African region. Two large epidemics were stopped and declared as ended (Highly Pathogenic Avian Influenza (HPAI) 15 January to 19 August 2017; Marburg 19^th^ October - 8^th^ December 2017). The others appeared singly and sporadically always contained at the source. Expenditure data, only available for the Marburg outbreak, indicates that Uganda spent Shillings 5.2 billion equivalent to $ 1.0 million (2018-dollar value). Uganda has stopped and contained human trypanosomiasis outbreaks, previous Ebola outbreaks and these other recent outbreaks at the source by applying a multi sectoral One Health (OH) approach with limited funding.^20-22^ Okello and others have also shown the efficiency and effectiveness of multisectoral coordination mechanisms in preventing and controlling health threats in Nigeria, Tanzania and Uganda.^23^ The Uganda Emergency Operations Center (EOC) estimates that a multisectoral OH rapid response team, comprising a veterinary officer, case management physician, social mobiliser, wildlife officer, environmental health officer and epidemiologist using 2 vehicles and drivers to respond to an outbreak within.

**FIGURE 5.**
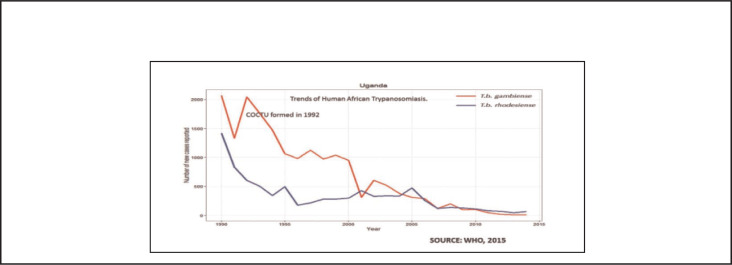
Trends of Human African Trypanosomiasis, Uganda 1990 to 2015

In Uganda, the frequency and case fatality caused by epidemics of human trypanosomiasis have rapidly declined following the establishment of the Coordination Office for the Control of trypanosomiasis (COCTU) in 1992^21-22^ (Figure 5).

A 3-pronged multisectoral strategy of (1) controlling the vector using both traps and spraying of animals (animal interventions), (2) reducing pathogen density by identifying and treating infected animals, and (3) identifying and treating human cases (human interventions) was applied. Trypanosomiasis is on the verge of elimination in Uganda 27 years after introducing the preventive strategy. Policy and decision makers can learn from the long-term investment (27 years) and long-term maintenance of routine activities for prevention, surveillance, case detection and treatment, with community engagement.

At the global level, major achievements in preventive efforts against human and animal diseases have been the eradication of small-pox and rinderpest, respectively.^30,31^

## RECOMMENDATIONS

### Adequate National Budget Allocation

The global community (development agencies, philanthropists and donors) need to work with the WHO and OIE to urge African countries to honour their commitments to invest in health systems strengthening, with stronger ownership and leadership reducing dependency on foreign aids. Allocating 15% of their budget for health and 1% of their GDP for research would allow institutionalisation of capacities built by projects and networks and boost efforts addressing gaps identified through the Joint External Evaluation (JEE) and highlighted by the 2014 Ebola outbreak.

### Establishment of a Regional Health Emergency Prevention Fund (RHEPF)

Regional governments facing common threats like the East African community under threat from hemorrhagic syndromes like Ebola, Riff valley fever, Marburg and Crimean Congo Hemorrhagic fevers bear responsibility and motivation to create a regional Prevention Fund aimed at containing disease outbreaks at the source. Ebola should also motivate African countries as a region to create such a fund. An RHEPF has the potential of reducing substantially the overall cost of response, as demonstrated by the case of Uganda.

National multisectoral coordination and collaboration mechanisms provide opportunities to monitor and thoroughly document the results and how funds are utilised. Mechanisms for servicing the fund should be explored, including member states contributions. This is an opportunity to build the essential backbone of the Africa CDC. Donors and development aid agents may set complementary levels supporting governments' contributions as part of strengthening One Health approach institutionalisation for transparency and accountability. The fund will invigorate Africa CDC's activities and strengthen its role of providing technical assistance and leadership on innovative strategies for preventing and controlling diseases in Africa. The regional fund may operate through open competitive calls, with clear eligibility criteria in order to maintain quality, strengthen national preparedness capacity, ownership and allow monitoring and documenting achievements over time.

### Global Health Emergency Prevention (GHEP) Fund

We recommend expanding the scope of the existing global health emergency funds including the UN Central Emergency Response Fund (CERF-2006), The World Bank Pandemic Emergency Financing Facility (PEF), and the WHO's Contingency Fund for Emergencies (CFE) to include financing preparedness capacity building. Their current scope is limited to financing countries in crisis and does not catalyze investing in routine preparedness capacity building.

## CONCLUSION

African governments bear the responsibility of protecting their populations from health threats and, therefore, cannot afford to wait for another Ebola-like event or to neglect the looming threat of antimicrobial resistance. Realising a new public health order for African health security requires investing in preparedness to shield Africa from the catastrophic consequences of uncontrolled health threats.^32^ Africa awaken!
